# ‘SASADECC’—A Unique Model of Collaborative Care Between Government and Non‐Government Health Services for Adolescents With Complex Substance Use Issues

**DOI:** 10.1111/dar.70109

**Published:** 2026-03-09

**Authors:** David E. A. Gordon, Belinda Volkov, Arezu Akbarian, Lauren A. Monds, Mark Montebello

**Affiliations:** ^1^ NSLHD Drug and Alcohol Services Sydney Australia; ^2^ Specialty of Addiction Medicine, Northern Clinical School The University of Sydney Sydney Australia; ^3^ Sydney Drug Education and Counselling Centre Sydney Australia; ^4^ NSW Drug and Alcohol Clinical Research and Improvement Network Sydney Australia; ^5^ National Drug and Alcohol Research Centre UNSW Sydney Sydney Australia

**Keywords:** adolescent, collaborative care, drug and alcohol, mental health, youth

## Abstract

**Introduction:**

Despite the importance of early intervention, major treatment gaps exist for adolescents with substance use issues. This report describes a unique collaboration between government and non‐government health services—The Specialist Addiction Service for Adolescents (SASA) adolescent addiction psychiatry service and Sydney Drug Education and Counselling Centre youth alcohol and other drug counselling service.

**Methods:**

The collaboration developed organically from available resources and close collaboration between the services. Informal client and referrer feedback, demographic data, outcome measures and clinical notes (2019–2022) were reviewed.

**Results:**

Feedback on the collaboration was positive overall. There were 149 Sydney Drug Education and Counselling Centre clients seen by SASA during the study period. Of these, 50 study participants were identified with valid serial outcome measures (VSOM) for analysis. Study participants did not differ significantly in demographics and primary drug of concern compared to all clients (with and without VSOM) combined. Overall, participants showed statistically significant improvements between entry and exit scores for the Severity of Dependence Scale, and for the Depression Anxiety Stress Scales—Short Form (DASS21) depression and the anxiety subscales, but not for the stress subscale. The majority of participants reported improvements towards substance use goals and psychological function. Many also improved in other areas of concern including social‐occupation function and risk issues.

**Discussion and Conclusions:**

The collaboration provides a useful model of collaborative care between government and non‐government services for adolescents with substance use issues. This paper reflects on learnings from the model for consideration in future service developments.

## Key Point

1. The collaboration provides a model of a specialist alcohol and other drug (AOD) service for adolescents.
2. The collaboration provides a model of collaborative care between government and non‐government AOD services.
3. Selective referral by AOD counsellors to psychiatric assessment and interventions can help clients engaged in AOD counselling where underlying mental health issues are impacting treatment progress, as demonstrated by improvements in outcome measures, clinical notes and feedback from clients and referrers.

## 1 Introduction

Adolescent substance use issues are common [1] and often complex [2]. Earlier onset of use increases the risk of problematic use, developmental impacts and harm [3, 4, 5]. Comorbid substance use and mental health issues are common, especially in younger clients of alcohol and other drug (AOD) services [6], with each impacting the severity and management of the other [7]. Managing these issues requires treatment approaches tailored for young people [8].

Historically, appropriate services to manage these issues have been lacking [9]. Adolescent health and mental health services are scarce [10] and lack expertise managing substance use issues. Government AOD services commonly exclude people under 18 years old. Non‐government organisations (NGO) addressing adolescent substance use typically lack psychiatric input. Vulnerable young people fall through treatment gaps.

The Sydney Drug Education and Counselling Centre (SDECC) is a community‐based NGO providing youth‐specific services for clients aged 12–25 years‐old for over 20 years including parents and carers. SDECC provides free counselling and support for AOD use with co‐occurring mental health issues, focusing on harm‐minimisation and wellbeing.

The Specialist Addiction Service for Adolescents (SASA) is a community adolescent addiction psychiatry consultation‐liaison service in the Northern Sydney Local Health District (NSLHD), founded in 2019 with a single child and adolescent addiction psychiatrist and later expanded with the addition of an addiction psychiatry advanced training registrar position.

This report reflects on ‘SASADECC’, the unique collaboration between SDECC and SASA, and implications for future directions in care for adolescents with substance use issues. Available client data, routine outcome measures and informal feedback from clients and referrers were pragmatically reviewed retrospectively with the aim of identifying what elements of the collaboration functioned well or not, as a pilot project to inform future service development and more formal research in the future.

## 2 Methods

### 2.1 Service Development and Feedback

Adolescent addiction psychiatry consultations began within a general addiction medicine outpatient clinic in 2018, offered only to NSLHD services and SDECC due to limited availability.

Informal client feedback was elicited by referrers following SASA input and, together with feedback from referrers themselves, was discussed with senior SDECC staff at SDECC case review and business meetings. Client and referrer feedback was then discussed between senior SDECC and SASA staff to identify barriers to successful referral and devise potential solutions to test in ongoing service development. Themes were identified in the feedback as a pilot project to inform a potential future formal qualitative study.

### 2.2 Review of Data

This project received Ethics approval (University of Sydney Human Research Ethics Committee, Project no. 2021/867).

Demographic data were collected from a convenience sample of all shared clients seen by SASA referred from SDECC between 2019 and 2022. Study participants included all shared clients who completed at least 2 rounds of valid serial outcome measures (VSOM) with SDECC – (i) ‘intake’ measures taken < 3 months before; and (ii) ‘exit’ measures < 3 months after the SASA episode of care.

#### 2.2.1 File Reviews

Medical records were reviewed retrospectively utilising SASA medical officers' case formulations, DSM‐5 [11] diagnoses and evidence of change in substance use, mental health, physical, social and risk issues as documented by the treating clinician. Risk to self was defined as risk of intentional self‐harm and suicide as assessed by SASA clinicians based on history and mental state examination. Risk of misadventure was similarly defined by clinicians as risk of unintentional injury including accident and overdose death, or vulnerability to intentional harm by others, typically due to substance effects such as reduced level of consciousness or incoordination. Misadventure does not include harm caused by other physiological effects of substances short of overdose.

#### 2.2.2 Outcome Measures

Clients completed the Severity of Dependence Scale (SDS) [12] assessing substance use and Depression Anxiety Stress Scales—Short Form (DASS21) [13] for mental health at a minimum of 3‐monthly intervals of treatment with SDECC.

Paired samples *t*‐tests compare intake and exit SDS and DASS21 scores. Chi‐square analysis delineates categorical changes in DASS21 scores.

## 3 Results

### 3.1 Service Development and Feedback

Client and referrer feedback identified key barriers: Attending a new service with a new clinician at a different location, colocation with adult services, inter‐service disconnection, unclear referral pathways, limited understanding of addiction psychiatry amongst referrers and inconsistent family inclusion models between services. Client engagement was impacted by readiness for change, substance use and mental health comorbidity and prior negative experiences with treatment, authority, stigma and trauma.

A senior SDECC clinician was appointed to assess referrals for appropriateness and priority, considering mental health needs, case complexity, risk, counselling barriers, readiness and attendance commitment. Offering SASA consultations at SDECC's rooms provided familiar, age‐appropriate facilities. Colocation facilitated SASA input to SDECC case review meetings and education program, increasing informal collaboration, education and clinician mutual support. Including referrers and other supports in SASA consultations reduced barriers, minimised handover time costs and leveraged rapport and clinician credibility already established by SDECC counsellors. Referrers could better prepare clients for psychiatric assessments and reinforce understanding of psychiatric formulations and management plans.

Ongoing referrer feedback highlighted the success of developmentally appropriate, client‐centred, non‐judgemental and trauma‐informed engagement; prioritising rapport‐building and flexible pragmatism; representing an alignment of values and synergy between services. Low rates of non‐attendance to SASA appointments were noted in feedback from referrers, who credited this to effective client selection and strategies employed to reduce barriers. Referrers reported that colocation and integration of services created a sense of camaraderie and mutual support among involved clinicians that was particularly beneficial when working with clients with complex needs.

### 3.2 Review of Data

During 2019–2022 SDECC saw 798 clients across 1215 episodes of care. Of these, 149 were seen by SASA; 50 study participants were identified with VSOM. Demographic data for one participant was partially missing.

Demographics, primary drug of concern (PDOC) and reason for treatment cessation are presented in Table 1. Demographics and PDOC of participants were not significantly different to all shared clients combined (all *p* > 0.05).

**TABLE 1:** Demographic data, primary drug of concern, injecting drug use and reason for treatment cessation of participants compared to all clients shared by the Sydney Drug Education and Counselling Centre and Specialist Addiction Service for Adolescents (SASA) during 2019–2022.

	Category	Counts of all clients	% of all clients	Number of participants	% of participants	Chi‐square (χ^2^)	df	*p*
Age of commencement with SASA	14	2	1.3%	0	0%	14.2	12	0.289
	15	9	6.0%	4	8%			
	16	22	14.8%	5	10%			
	17	18	12.1%	7	14%			
	18	23	15.4%	5	10%			
	19	19	12.8%	8	16%			
	20	7	4.7%	5	10%			
	21	11	7.4%	3	6%			
	22	19	12.8%	4	8%			
	23–26	19	12.8%	9	18%			
Sex	Female	71	47.7%	26	52%	0.345	1	0.557
	Male	78	52.3%	24	48%			
Country of birth	Australia	119	83.2%	41	84%	14.1	10	0.167
	Other	24	16.8%	8	16%			
Indigenous status	Neither Aboriginal/Torres Strait Islander	136	95.1%	45	92%	3.63	3	0.304
	Unknown	4	2.8%	3	6%			
	Aboriginal origin	2	1.4%	1	2%			
	Declined to respond	1	0.7%					
Preferred language	English	136	95.1%	47	96%	6.41	4	0.17
	Other	7	4.9%	2	4%			
Principal source of income	Dependent on others	70	47.0%	22	44%	4.78	7	0.686
	Part‐time employment	36	24.2%	13	26%			
	Full‐time employment	18	12.1%	7	14%			
	Temporary benefit (e.g., unemployment)	14	9.4%	5	10%			
	No income	3	2.0%	1	2%			
	Other	3	2.0%	2	4%			
	Not stated/not known/inadequately described	3	2.0%	0	0%			
	Pension (e.g., aged, disability)	2	1.3%	0	0%			
Living arrangements	Parent(s)	119	79.9%	38	76%	4.65	5	0.46
	Other	9	6.0%	5	10%			
	Friend(s)	9	6.0%	4	8%			
	Spouse/partner attention‐deficit hyperactivity disorder	6	4.0%	1	2%			
	Alone	4	2.7%	2	4%			
	Other relative(s)	2	1.3%	0	0%			
Usual accommodation	Rented house or flat (public or private)	86	57.7%	30	60%	5.54	3	0.136
	Privately owned house or flat	56	37.6%	15	30%			
	Hostel/supported accommodation service	6	4.0%	4	8%			
	Other	1	0.7%	1	2%			
Primary drug of concern	Cannabinoids and related drugs	97	65.1%	32	64%	11.9	13	0.534
	Alcohol	30	20.1%	13	26%			
	Stimulants	9	6%	1	2%			
	Opioids	5	3.4%	1	2%			
	Benzodiazepines	5	3.4%	1	2%			
	Nil or inadequately described	3	2.0%	2	4%			
Injecting drug use	Never injected	138	92.6%	50	100%	5.15	4	0.272
	Ever injected	9	6%	0	0%			
	Not stated/inadequately described	2	1.3%	0	0%			
Reason for cessation	Service completed	101	67.8%	37	74%	12.8	4	0.012
	Left without notice	34	22.8%	5	10%			
	Transferred/referred to another service	8	5.4%	6	12%			
	Moved out of area	5	3.4%	2	4%			
	Other	1	0.7%	0	0%			

The majority of clients were Australian‐born, non‐indigenous and primarily spoke English. Most lived with parents in rented accommodation; approximately half were dependent on others, and 10% received government benefits as their primary income.

Nearly 30% of study participants identified as sexuality‐ or gender‐diverse, though clients were not necessarily routinely asked as these questions are not currently included in NSW Health's Minimum Data Set.

PDOC were predominantly cannabinoids, then alcohol. File reviews identified 63% drinking at harmful levels, 61% with nicotine use disorder and 60% having polysubstance use issues (using at least three different substances, excluding nicotine and caffeine). As per Table 1, overall few clients referred to SASA by SDECC reported ever having injected drugs, including none of the study participants with VSOM.

The majority of clients completed the service, while 22.8% left without notice; completers were more likely to have VSOM.

#### 3.2.1 File Reviews

On review of client files, all participants had identified substance use disorders, with 76% reporting improvement towards their substance use goals. Mental health diagnoses were common (98%), with 72% of these showing improvement documented by the treating clinician. Physical health issues affected 31%, with 81% noted to improve. Social issues were widespread (98%)—including family (88%), peer (80%) and occupational (63%) issues—and 70% of clients with identified social issues were reported to experience improvement in these areas. Significant risk to self or of misadventure was identified in > 70%, improving in 72%. Trauma history was noted in 90%; PTSD was the most common diagnosis (53%), often co‐occurring with borderline personality disorder (24%). Generalised and social anxiety each appeared in ~20% but were also often subsumed under PTSD. Cannabis use was commonly noted in SASA clinicians' clinical formulations to have triggered or exacerbated social anxiety symptoms in a dose‐dependent relationship.

Attention‐deficit hyperactivity disorder and autism assessments were not provided, though attention‐deficit hyperactivity disorder had been previously diagnosed in 33% and suspected in a further 12% of clients; autism was previously diagnosed in 8% and suspected in a further 8%.

Management recommendations included lifestyle measures (100%), psychological strategies (96%) and medication changes (80%).

#### 3.2.2 Outcome Measures

Statistical analysis of serial outcome measures is presented in Table 2.

**TABLE 2:** Serial outcome measures of participants.

Outcome measure	Statistic	Change	Count	Proportion	*χ* ^2^	df	*p*	Mean difference	SE difference	Effect size (Cohen's *d*)	*N*	Mean	Median	SD	SE
SDS intake score	Student's t					3.91	54	< 0 0.001	2.18	0.558	0.527	55	8.42	9	3.41
SDS exit score	Student's t					3.91	54	< 0 0.001	2.18	0.558	0.527	55	6.24	7	4.28
DASS21 depression intake score	Student's t					4.3	58	< 0 0.001	3.17	0.738	0.559	59	11.86	13	5.66
DASS21 depression exit score	Student's t					4.3	58	< 0 0.001	3.17	0.738	0.559	59	8.69	8	5.36
DASS21 depression change	*χ* ^2^	No change	29	0.527	14.7	2	< 0 0.001								
	*χ* ^2^	Positive change	20	0.364											
	*χ* ^2^	Negative change	6	0.109											
DASS21 anxiety intake score	Student's t					4.05	58	< 0 0.001	2.36	0.582	0.527	59	10.54	11	4.52
DASS21 anxiety exit score	Student's t					4.05	58	< 0 0.001	2.36	0.582	0.527	59	8.19	8	4.18
DASS21 anxiety change	*χ* ^2^	No change	20	0.364	12.3	2	0.002								
	*χ* ^2^	Positive change	28	0.509											
	*χ* ^2^	Negative change	7	0.127											
DASS21 stress intake score	Student's t					1.81	58	0.076	1.08	0.6	0.235	59	12.51	13	4.16
DASS21 stress exit score	Student's t					1.81	58	0.076	1.08	0.6	0.235	59	11.42	11	4.3
DASS21 stress change	*χ* ^2^	No change	39	0.7091	37.7	2	< 0 0.001								
	*χ* ^2^	Positive change	13	0.2364											
	*χ* ^2^	Negative change	3	0.0545											
DASS21 total intake score	Student's t					4.44	58	< 0 0.001	6.61	1.488	0.578	59	34.92	37	11.84
DASS21 total exit score	Student's t					4.44	58	< 0 0.001	6.61	1.488	0.578	59	28.31	27	11.96
DASS21 total score change	*χ* ^2^	No change	2	0.0345	49.8	2	< 0 0.001								
	*χ* ^2^	Positive change	44	0.7586											
	*χ* ^2^	Negative change	12	0.2069											

Abbreviations: DASS21, Depression Anxiety Stress Scales – Short Form; SDS, Severity of Dependence Scale.

Significant improvements were observed in SDS scores (*t*(54) = 3.91, *p* < 0.001); and DASS21 Depression (*t*(58) = 4.30, *p* < 0.001), Anxiety (*t*(58) = 4.05 *p* < 0.001) and Total scores (*t*(58) = 4.44, *p* < 0.001). No significant difference was found in DASS21 Stress scores (*t*(58) = 1.81, *p* = 0.076).

Chi‐square analysis of DASS21 scores indicated significant differences in the distribution of changes amongst participants, with a general trend towards positive outcomes. Positive, nil and negative changes were recorded as follows: for Depression, 36.4%, 52.7% and 10.9% (*χ* ^2^ = 14.7; *p* < 0.001); for Anxiety, 50.9%, 36.4% and 12.7% (*χ* ^2^ = 12.3; *p* = 0.002) and for Stress, 23.6%, 70.9% and 5.5% (χ^2^ = 37.7; *p* < 0.001). Overall, 76% of participants showed positive changes in total DASS21 scores (χ^2^ = 49.8; *p* < 0.001), indicating significant mental health improvements, with minimal negative changes across all measures.

## 4 Discussion

The analysis suggests that collaboration effectively reduced substance use severity, depression and anxiety levels in clients selected by SDECC clinicians. However, significant limitations include the pragmatic, retrospective design and absence of a control group.

Participant numbers were limited by the methodology of pragmatically utilising SDECC‐collected data retrospectively, where there were issues with the consistency of outcome measure collection or the timing of routine outcome measures may not have fit with the timing of SASA input.

Whilst 149 clients attended SASA, 798 clients were seen by SDECC in the same period. This might be considered a low rate of referral given the high rate of mental health comorbidity typically seen in AOD service client populations. Whilst some of this gap may be due to clients already receiving care in public and private mental health services, and others declined or were otherwise unready to accept referral, ongoing work is clearly required to address significant treatment gaps and barriers.

It is important to note that clients were selected for referral often because mental health issues were identified as barriers to progress with their substance use goals, but also conversely where mental health issues persisted despite managing substance use. Those with mental health issues impeding substance use management came to SASA with more room for improvement in scores for both outcome measures assessing substance use (SDS) and mental health (DASS21). Those with residual mental health issues despite addressing substance use were often noted by referrers to have already made significant improvements in both outcome measures prior to being referred to SASA.

The colocation of adolescent, youth and adult services and the need for environments tailored to young people's needs remains an issue. The collaboration attempted to address this by establishing SASA clinics within SDECC's youth‐friendly facilities. However, limited resourcing and systems issues around staffing and training regulations for registrars pose barriers to the expansion of this service solution.

SASA treatment completion rates were high (67.8%) and loss‐to‐follow‐up rates low (22.8%), outperforming estimated average dropout rates for outpatient (non‐opioid) substance use treatment programs of approximately 30% within the first month and over 50% within three months [14]. High diagnosis rates reflect effective referral selection by SDECC clinicians, without the barrier of requiring a general practitioner referral to the psychiatrist.

The prevalence of trauma and significant risk highlights the importance of trauma‐informed care and the need to support clinicians managing vicarious trauma. Collaborative care provides mutual benefits for SDECC and SASA clinicians through shared professional support.

Comorbid mental health, social issues, neurodiversity and sexuality‐ and gender‐diverse presentations were prominent, warranting tailored consideration in future service development. Informal client feedback was well‐captured through the overlapping service structure, though more formal consumer input remains an area for improvement. The identification of strong themes in the feedback begs the development of future formal qualitative research to inform ongoing service development.

The common observation by SASA clinicians of a dose‐dependent relationship between cannabis use and social anxiety symptoms is worthy of further research examination.

Further service development including a prospective research design with tailored outcome measures and active follow‐up is required to corroborate these pilot findings.

Sustainability and scalability of the service are limited by specialist availability. Despite the high prevalence of comorbid AOD and mental health issues in Australia [7, 15], opportunities for psychiatry trainees to gain experience in AOD services and harm minimisation remain scarce.

Learnings from the collaboration have informed the development of the new AOD Consultation, Assessment, Care and Intervention for Adolescents (ACACIA) Service. ACACIA offers multidisciplinary team care and case management for adolescents aged 12–24 with moderate‐to‐severe biopsychosocial issues related to problematic substance use in the Northern Sydney Local Health District.

ACACIA now supports a Royal Australian and New College of Psychiatrists‐accredited advanced training position in addiction and child and adolescent psychiatry.

ACACIA continues to collaborate closely with SDECC including to identify ongoing treatment gaps and barriers for young people with issues related to problematic substance use.

## 5 Conclusion

The ‘SASADECC’ model demonstrates effective collaboration between government and NGO services, emphasising the value of strong referral pathways while delivering flexible, individualised and client‐centred care.

For most SDECC clients, psychiatric care would otherwise remain inaccessible, as AOD issues often complicate engagement with traditional mental health services. SDECC clinicians' capacity to establish safety and rapport facilitated effective psychiatric referrals, reducing barriers and optimising the use of limited resources like SASA.

The synergy and camaraderie between these highly collaborative services created a safety net that helped prevent vulnerable young people from falling through systemic gaps.

## Author Contributions

Each author certifies that their contribution to this work meets the standards of the International Committee of Medical Journal Editors.

## Funding

The authors have nothing to report.

## Conflicts of Interest

The authors declare no conflicts of interest.

## Data Availability

The data that support the findings of this study are available on request from the corresponding author. The data are not publicly available due to privacy or ethical restrictions.
